# Diagnosis of Breast Hyperplasia and Evaluation of RuXian-I Based on Metabolomics Deep Belief Networks

**DOI:** 10.3390/ijms20112620

**Published:** 2019-05-28

**Authors:** Mingyang Jiang, Yanchun Liang, Zhili Pei, Xiye Wang, Fengfeng Zhou, Chengxi Wei, Xiaoyue Feng

**Affiliations:** 1 Key Laboratory of Symbolic Computation and Knowledge Engineering of the Ministry of Education, College of Computer Science and Technology, Jilin University, Changchun 130012, China; mingyangjiang@imun.edu.cn (M.J.); ycliang@jlu.edu.cn (Y.L.); ffzhou@jlu.edu.cn (F.Z.); 2 Inner Mongolia Engineering Research Center of Personalized Medicine, College of Computer Science and Technology, Inner Mongolia University for the Nationalities, Tongliao 028000, China; zhilipei@imun.edu.cn; 3 Zhuhai Sub Laboratory, Key Laboratory of Symbolic Computation and Knowledge Engineering of the Ministry of Education, Zhuhai College of Jilin University, Zhuhai 519041, China; 4 College of Chemistry and Chemical Engineering, Inner Mongolia University for the Nationalities, Tongliao 028000, China; xywang@imun.edu.cn; 5 Inner Mongolia Key Laboratory of Mongolian Medicine Pharmacology for Cardio Cerebral Vascular System, Medicinal Chemistry and Pharmacology Institute, Inner Mongolia University for the Nationalities, Tongliao 028000, China; cxwei@imun.edu.cn

**Keywords:** breast cancer, deep belief networks, Mongolian medicine, metabolomic data

## Abstract

Breast cancer is estimated to be the leading cancer type among new cases in American women. Core biopsy data have shown a close association between breast hyperplasia and breast cancer. The early diagnosis and treatment of breast hyperplasia are extremely important to prevent breast cancer. The Mongolian medicine RuXian-I is a traditional drug that has achieved a high level of efficacy and a low incidence of side effects in its clinical use. However, for detecting the efficacy of RuXian-I, a rapid and accurate evaluation method based on metabolomic data is still lacking. Therefore, we proposed a framework, named the metabolomics deep belief network (MDBN), to analyze breast hyperplasia metabolomic data. We obtained 168 samples of metabolomic data from an animal model experiment of RuXian-I, which were averaged from control groups, treatment groups, and model groups. In the process of training, unlabelled data were used to pretrain the Deep Belief Networks models, and then labelled data were used to complete fine-tuning based on a limited-memory Broyden Fletcher Goldfarb Shanno (L-BFGS) algorithm. To prevent overfitting, a dropout method was added to the pretraining and fine-tuning procedures. The experimental results showed that the proposed model is superior to other classical classification methods that are based on positive and negative spectra data. Further, the proposed model can be used as an extension of the classification method for metabolomic data. For the high accuracy of classification of the three groups, the model indicates obvious differences and boundaries between the three groups. It can be inferred that the animal model of RuXian-I is well established, which can lay a foundation for subsequent related experiments. This also shows that metabolomic data can be used as a means to verify the effectiveness of RuXian-I in the treatment of breast hyperplasia.

## 1. Introduction

In Cancer Statistics 2018, breast cancer was identified as the leading cancer type among the estimated new cases, and is the second most frequent type among estimated deaths in American women [1]. Core biopsy data have shown a close association between breast hyperplasia (BH) and breast cancer (BC) [2]. Breast lesions can be either benign or malignant, and the probability of a lesion being benign is higher. BH is one of the leading causes of breast lesions, and refers to hyperplasia of the mammary epithelium and fibrous tissue, degenerative lesions in the mammary ducts and lobules, and the progressive growth of connective tissue [3,4]. The pathogenesis of BH is mainly due to the imbalance of endocrine hormones. Therefore, the early diagnosis and treatment of a BH is extremely important.

Glasl et al. [5] have found that in specific diseases, such as chronic diseases, head trauma, and diseases of the liver, stomach, and kidneys, Mongolian medicine has good therapeutic efficacy and has gained more attention in drug development. The Mongolian medicine RuXian-I, which has a high efficacy and low incidence of side effects, is currently in clinical use as a traditional drug. However, based on metabolomic data, a rapid evaluation method for the efficacy of RuXian-I is still lacking [6]. Metabolomic methods have great potential in evaluating the therapeutic effects of traditional Mongolian medicine. As an emerging approach, metabolomic methods have received more attention in recent years. Metabolomics research uses quantitative methods to describe the changes, species, quantity, and variation of the expression levels of bio-endogenous small-molecule metabolites. The goal of metabolomics is to identify metabolites that distinguish groups of experimental sample data from the control groups (CGs), treatment groups (TGs), and model groups (MGs), where the MGs are different from the CGs in that they are affected by factors such as diseases, drugs, and poisons, as well as environmental, genetic, or physiological factors [7,8]. Metabolite profiling can be used to identify metabolites and physiological states in metabolomics studies, ultimately to understand the underlying biological mechanisms [9,10]. Therefore, a metabolomics profile analysis is one of the most important facets of metabolomic research [11,12].

Most metabolomic data are obtained by gas chromatography time-of-flight mass spectrometry (GC-TOF-MS), ultra-high-performance liquid chromatography (UPLC-MS), and nuclear magnetic resonance (NMR). They must be pre-processed for further statistics and analysis. When metabolomic data are obtained by spectral or mass spectrometry, the amount and complexity of data are increased with the improvement in the precision and functionality of the instrument. Metabolomic data have hundreds to thousands of variables, but only a small portion of the data is associated with the physiological state, while much of the remaining data are noise [13]. The analysis meets new difficulties and challenges due to its high throughput, and sparse and high-dimensional nature [14,15]. 

Many machine learning algorithms have been applied to metabolomic data analysis. For example, Lin et al. [16] adopted combinational methods using support vector machine (SVM), genetic algorithm (GA), and random forest (RF) to extract metabolomic markers. Wang et al. [17] used a back propagation neural network algorithm (BPNN) to study the therapeutic mechanism of amomum compactum in gentamicin-induced acute kidney injury in rats. However, traditional machine learning algorithms cannot achieve satisfactory results for the metabolomic data of high-dimension, sparse, non-linear, and small data [18,19,20,21]. Bewick et al. [22] suggested that substantial sample data are needed when a logistic regression algorithm is used to solve the metabolomic data classification problem. Moreover, if the relationships among the variables are incorrectly extracted when the feature selection method is explored before classification, the classification result will be of low accuracy [23,24]. Therefore, with metabolomic data, a more effective and efficient model is needed to extract features.

In 2006, Hinton et al. [25] proposed deep belief networks (DBN) and demonstrated their effectiveness in many applications, such as handwritten digits recognition and natural language understanding. Their research laid the theoretical foundation for the application and development of deep artificial neural networks. Deep learning is one of the most representative feature extraction methods in the machine learning field [26]. It can extract high-level abstract features from heterogeneous and high-dimensional data sets, such as metabolomic data sets [27]. The deep learning approach has been applied successfully in many areas, such as image processing, audio processing, video processing, natural language understanding, and bioinformatics. Cireşan et al. [28] proposed biologically plausible deep artificial neural network architectures that can match human performance in tasks such as the recognition of handwritten digits or traffic signs. Hinton et al. [29] used an improved DBN algorithm to compress a document, and this can be used for document retrieval. Huang et al. [30] proposed a new speech emotion recognition method based on DBN and SVM, using DBN to extract emotional features in speech signals automatically. The features, as the training result of DBNs, were the input of a nonlinear SVM classifier. Putin et al. [31] designed a modular ensemble of 21 deep neural networks (DNNs) of varying depth, structure, and optimization to predict human chronological age using a basic blood test. Although deep learning models have achieved many exciting results in the aforementioned research areas, to the best of our knowledge, there is no related study assessing the hyperplasia of mammary glands.

Using experimental data from female Wistar rats, we introduced a framework, named the metabolomics deep belief network (MDBN), to combine deep belief networks and softmax regression for the diagnosis of BH and the evaluation of RuXian-I. In the process of training, the unlabelled data were used for pretraining, and then the labelled data were used to fine-tune based on a limited-memory Broyden Fletcher Goldfarb Shanno (L-BFGS) algorithm [32]. To avoid overfitting, the dropout strategy was added during the training procedures. We used the hybrid algorithm to perform the classification of the metabolomic data of positive or negative spectra, and compared the results with SVM, k-nearest neighbors algorithm (KNN), and BPNN. The five-fold cross-validation was used to complete the classification experiment. In each fine-tuning process, the mean square error (MSE), and misclassification rates of training data and the test data were recorded. 

## 2. Results

**Figure 1:**
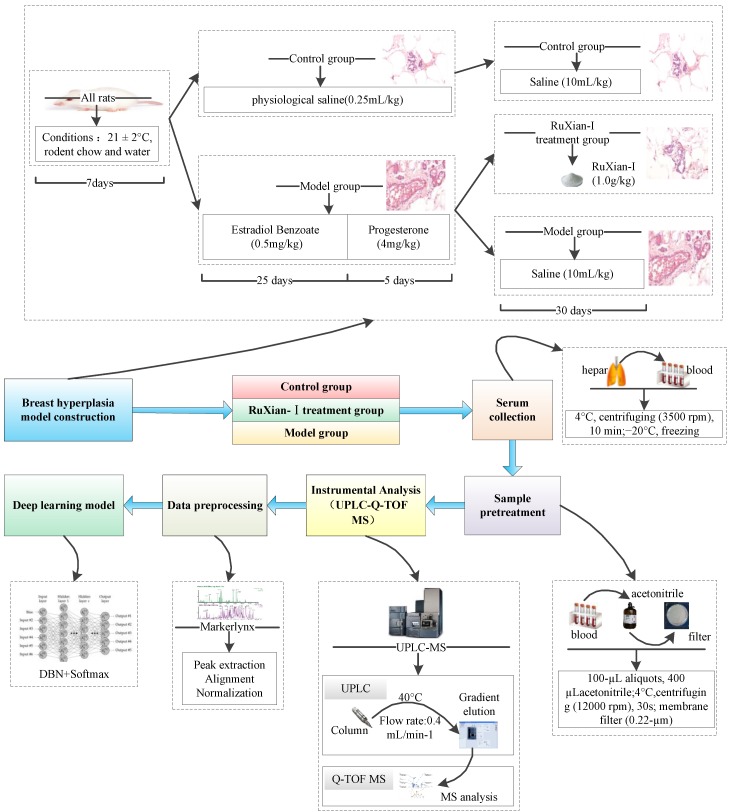
The framework of the metabolomics deep belief network (MDBN).

In this section, we describe the data set and our experimental results for the diagnosis of BH and evaluation of RuXian-I based on MDBN. The first part describes how to obtain the metabolomic dataset we used. In the second part, we evaluated our proposed method, MDBN, in the metabolomic dataset. The framework of MDBN is shown in Figure 1.

### 2.1. Dataset

#### 2.1.1. Chemicals and Reagents

Herein, oestradiol benzoate and progesterone were obtained from the Shanghai Tongyong Pharmaceutical Company Ltd., Shanghai, China. Methanol and formic acid (Fisher Scientific, Leicestershire, UK) were of HPLC grade. Distilled water was obtained from A. S. Watson Group Ltd. (Hongkong, China). RuXian-I was provided by the Mongolian Medicine Manufacturing Room of the Affiliated Hospital of Mongolia University for the Nationalities. The components of RuXian-I are listed in Ref. [33].

#### 2.1.2. Breast Hyperplasia Model Construction and Treatment

As shown in Figure 1, there were 168 female Wistar rats (bodyweight, 220 ± 10 g) provided by the Affiliated Hospital of Inner Mongolia University for the Nationalities. They were randomly divided into three groups comprising fifty-six rats each: CG, MG, and the RuXian-I treatment group (RG). All the animals were reared under standard conditions (21 ± 2 °C) with free access to rodent chow and water, and they were allowed to acclimatize in metabolism cages for 1 week prior to experimentation. The rats in the CG were injected with normal saline (0.25 mL/kg) for 30 days. The disease model rats were injected with oestradiol benzoate (0.5 mg/kg) for 25 days, followed by progesterone (4 mg/kg) for 5 days. Then, they were randomly divided into two groups (fifty-six rats in each group)—MG and RG. The rats in the RG group were maintained on RuXian-I (1.0 g/kg, oral gavage) for 30 days. The rats in the CG and MG groups were given saline (10 mL/kg, oral gavage) for 30 days. Blood was collected from different groups from the hepatic portal veins and then was centrifuged at 3500 rpm for 10 min at 4 °C. The supernatants were frozen immediately, and then they were stored at −20 °C and thawed before analysis.

The serum samples were thawed, and 100 µL aliquots were added to 400 µL acetonitrile. The mixtures were vortexed for 30 s and centrifuged at 12,000 rpm for 10 min at 4 °C. A 0.22 µm membrane filter was used to filter the supernatant.

#### 2.1.3. UPLC-MS Conditions

A Waters Acquity UPLC system coupled with a quadrupole time-of-flight Xevo G2-S mass spectrometer (Waters, UK) was used for the metabolomic analysis. A Waters ACQUITY UPLC BEH C18 Column (1.7 µm, 2.1 × 50 mm, Waters, USA) kept at 40 °C, with a flow rate of 0.4 mL/min^−1^, was used for the separation. Formic acid (0.1%) in deionized water (A) and methanol (B) was used as the mobile phase. The gradient elution of B was as follows: 8–80% B at 0–3 min, 80–100% B at 3–6 min, 100% B at 6–8 min, 100–8% B at 8–9 min, and then it was kept constant at 8% B for 2 min. The sample injection volume was 5 µL.

The electrospray ion source was used in both the positive and negative ion mode in the MS analysis. In the positive ion mode, the source temperature was 150 °C, and the desolvation gas temperature was 400 °C. The capillary, cone, and offset voltages were 3.2 kV, 35 V, and 70 V, respectively. In the negative ion mode, the source temperature was 95 °C, and the desolvation gas temperature was 190 °C. The capillary, cone, and offset voltages were 2.6 kV, 40 V, and 80 V, respectively.

#### 2.1.4. Data Analysis

MarkerLynx Application Manager software in Masslynx V4.1 was used to process the UPLC-MS raw data files. After peak detection, peak alignment, and data normalization, the data matrix was established. To obtain adequate information regarding the metabolites, both positive and negative spectra were applied in the mass spectrometry.

### 2.2. Classification Experiment

#### 2.2.1. Classification Experiment of the Positive Spectrum Data

To verify the reliability and stability of the MDBN framework, the experiment adopted a five-fold cross-validation method due to the small number of samples in the metabolomic data set. There were 134 samples in the training set, 34 samples in the test set, and 2889 variables for the positive spectral data. The structure of our neural network was 2889-500-100-3. First, the unsupervised learning method was used to complete the pretraining of the DBN model. After the initial weights were obtained, the supervised learning method was used to train the softmax regression. Finally, the gradient descent algorithm (GD) and L-BFGS were used to fine-tune the system model. Due to the small amount of training and test data, we did not divide the data into min-batches during the experiment. For the five-fold cross-validation method, all the data were divided into an average of five groups. Four groups were selected as the training set each time, and the remaining group was the test set. This process was repeated until each group became a test set. In the DBN training process, the number of iterations was 500 for each restricted Boltzmann machines (RBM) unit. The classification accuracy is shown in Table 1. The BPNN classification accuracy was between 73% and 80% for the different groups. The KNN classification accuracy was 58.82% for the first group and between 79% and 86% for the other groups. The SVM classification accuracy was 85.29% for the third group, and the accuracy was between 61% and 68% for the other groups. When we used the combination method of DBN and softmax regression, in which fine-tuning was based on GD or L-BFGS, the classification accuracy was more than 88% for each group, and the accuracy did not fluctuate dramatically. 

In the classification experiment, two different fine-tuning methods were used in the MDBN. Regarding the classification results, although the classification accuracy of MDBN was obviously better than those of the other three methods for different groups of data, the classification results of the different fine-tuning methods were obviously different. The classification accuracy of DBN+L-BFGS+Softmax was obviously better than that of DBN+GD+Softmax. From the above analysis, as the number of iterations increases, the classification method based on DBN and Softmax is superior to that of the other three methods. From the classification results and the fine-tuning process, the proposed DBN method based on L-BFGS is superior to that of the DBN method based on GD. Therefore, the proposed method is more stable, reliable, and suitable for the classification of the BH metabolomic data.

**Figure 2:**
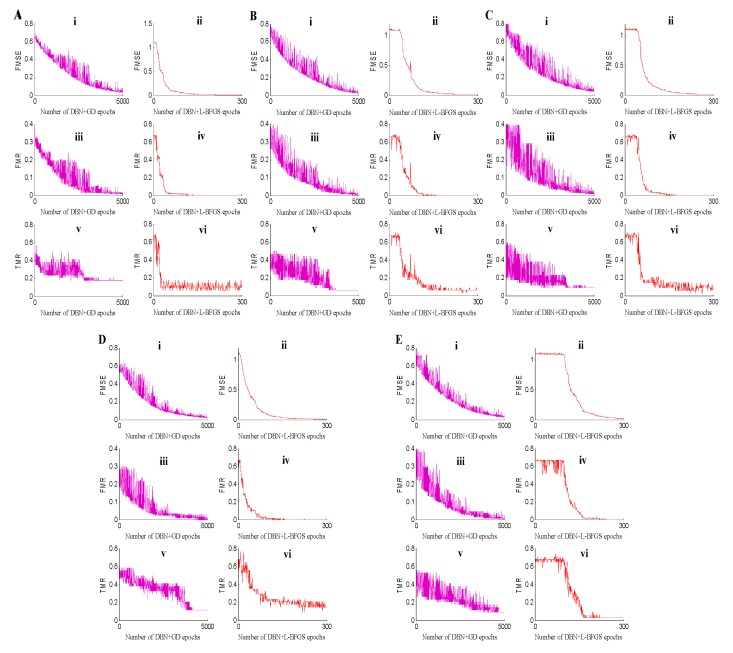
Fine-tuning experimental results on the five-fold data sets. In each subgraph of (**A**–**E**), (i) is the fine-tuning error mean square error (FMSE) of DBN+GD+Softmax, (ii) is the fine-tuning misclassification rate (FMR) of DBN+GD+Softmax, (iii) is the test misclassification rate (TMR) of DBN+GD+Softmax, (iv) is the fine-tuning error MSE (FMSE) of DBN+L-BFGS+Softmax, (v) is the fine-tuning misclassification rate (FMR) of DBN+L-BFGS+Softmax, and (vi) is the test misclassification rate (TMR) of DBN+L-BFGS+Softmax.

For deep neural networks, searching for the minimum is of great importance. To verify the searching speed of the minimum and the stability of the proposed method for metabolomic data classification, the dropout strategy was introduced to the training procedure to avoid the overfitting problem [33]. In addition to the classification accuracy, we selected the MSE, and training and test classification error rates to further illustrate the stability for the BH metabolomic data analysis. The experimental results of the five-fold datasets are shown in Figure 2. From the five subgraphs, we can conclude that the convergent speed of DBN+L-BFGS is quicker than that of DBN+GD. DBN+L-BFGS can find the minimum when the iteration number equal to 200, while DBN+GD can obtain the minimum when it runs to 5000 epochs. Meanwhile, for each measurement, DBN+L-BFGS performs better and more stable than DBN+GD.

#### 2.2.2. Classification Experiment of the Negative Spectrum Data

To verify the reliability and stability of the negative spectrum data, we also used the five-fold cross-validation method. The scales of the training and test sets for negative spectrum data were the same as those for the positive spectrum data. Additionally, all negative spectrum data for the system model were pre-processed and normalized. Compared with the positive spectrum data, the structure and parameters of the system model were different due to the low dimensions of the negative spectral data. There were 353 variables for the negative spectral data, and the structure of our network was 353-100-20-3. In the process of DBN training, the number of iterations for each RBM unit was 100, and the training data were not divided into min-batches. In the process of fine-tuning, the number of iterations was 300. The results are shown in Table 2.

From the results of the different groups in Table 2, the classification accuracies of all the methods were mostly better than those of the positive spectrum data. The classification accuracy of DBN+GD+Softmax was the highest from the first group to the fourth, and the highest classification accuracy of the fifth group was obtained from KNN. The BPNN and DBN+Softmax were very stable for each group, and the classification results of KNN were less satisfactory for the second group. The classification results of SVM varied greatly compared with those of the other methods, and it was the most unstable one for the negative spectrum data. From the results of the mean in Table 2, DBN+GD+Softmax had the highest classification accuracy of 92.94%, SVM had the lowest accuracy of 78.23%, and the other methods were very close, at approximately 88.50%. 

#### 2.2.3. Classification Experiments across Different Training and Test Datasets for the Positive Spectrum Data

To analyze the data size effects, we performed eight experiments using different training and test sets. The comparison results for the five models are shown in Table 3.

Table 3 shows that when the training set number is 50 and the test set number is 118, the classification result of DBN+GD+Softmax is better than that of DBN+L-BFGS+Softmax. For the other seven different training and test sets (from 60 to 120), the classification results of DBN+L-BFGS+Softmax are better than those of DBN+GD+Softmax. Thus, if there is less training data, then fewer features can be obtained by the machine learning algorithm, and the classification accuracy of each method is low. With the increase in the amount of training data, the BPNN, KNN, DBN+GD+Softmax, and DBN+L-BFGS+Softmax can learn more features, and the classification accuracy can be improved. For example, the classification accuracy of DBN+L-BFGS+Softmax increased from 66.95% to 94.31%. However, the classification accuracy of SVM was low, with no significant improvement.

**Figure 3:**
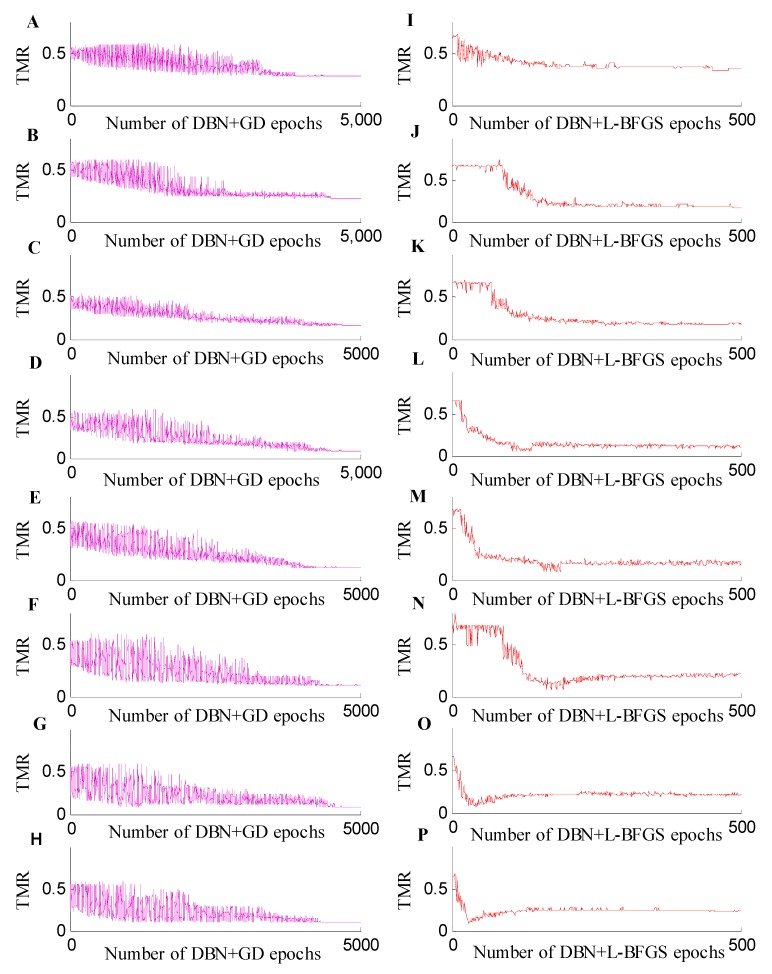
Misclassification rate curve from the 50 to 120 training sets. (**A**–**H**) are the training misclassification rate (TMR) curves of DBN+GD+Softmax for the number of training sets from 50 to 120, and (**I**–**P**) are the TMR curves of DBN+L-BFGS+Softmax for the number of training sets from 50 to 120.

For the classification experiments using different training and test sets, we also compared the classification accuracy of each fine-tuning (Figure 3). The RBM was used to obtain the initial weight of the DBN, and the system model was fine-tuned based on an L-BFGS algorithm. Although the amount of metabolomic data was small and high-dimensional, a stable model could be obtained. At the same time, the method proposed in this paper can produce better classification accuracy. The DBN+L-BFGS+Softmax reached an accuracy of 94.31%. In Figure 3, the horizontal axis represents the number of epochs, and the vertical axis represents the misclassification rate. It shows the curve of the test set misclassification rate, when the training sets were 50, 60, 70, 80, 90, 100, 110, and 120.

## 3. Discussion

In our previous study, Ru Xian-I was proven to be effective for breast hyperplasia by hematoxylin-eosin staining of mammary glands and an immunohistochemical experiment [33]. In this study, we proposed an MDBN to classify the BH metabolomic data. The results can aid the diagnosis of breast hyperplasia and further evaluations of the effectiveness of RuXian-I. 

In the classification experiment using the positive spectrum data, the SVM classification accuracy was different for each group, and the KNN method also lacked robustness because of its unstable accuracy. Although the results did not change significantly, the accuracy of the BPNN was not sufficient. The proposed MDBN generated a superior classification result that was both stable and reliable for the classification of the BH metabolomic data. In the classification experiment with the DBN models, for the DBN+GD+Softmax training process, the MSE, and training and test classification error rates in fine-tuning decreased gradually during oscillation, and the amplitude was found to be larger before stabilization. After reaching a steady state, the amplitude narrowed and tended to stabilize. However, the number of iterations for this process is larger, and the training time is longer. For the DBN+L-BFGS+Softmax training process, the values also gradually decreased during oscillation, but they decreased faster, the amplitude was smaller, and the number of iterations required to achieve stability was less. During fine-tuning, the number of iterations based on GD was 5000, and the number of iterations based on the L-BFGS algorithm was 300, such that the training time also obviously decreased.

In the classification experiment on the negative spectrum data, SVM was not suitable for the classification of BH metabolomic data, and KNN classification was sensitive for the different group data. The DBN was suitable for feature extraction or classification of high-dimensional data. The BPNN had a single hidden layer and fewer hidden nodes, and it was suitable for low-dimensional data. The DBN+Softmax and the BPNN models can produce closer and better classification results, and the two methods are stable and reliable in solving the metabolomic data classification problem.

In the classification experiments across different training and test datasets for the positive spectrum data, compared with the BPNN, KNN, and SVM, the DBN+Softmax model can achieve better classification results for different training and test sets. When the training establishment is larger than 70, the classification results of DBN+Softmax are stable, but the results are unstable for other methods. The DBN+GD+Softmax model is not stable at the initial stage of the fine-tuning process, and the classification error rate of the different scale test sets has a large amplitude. With the increase of iterations, the model tends to be stable and the amplitude of the error rate decreases, but it needs a much longer training time. For the DBN+L-BFGS+Softmax model, the classification error rate decreases rapidly, and it can obtain stable oscillation intervals approximately 100 times with a small oscillation amplitude. This means that the DBN+L-BFGS+Softmax model can find these minimums more quickly. In the classification experiments of different training and test sets, the DBN+GD+Softmax iteration number was 5000, and the DBN+L-BFGS+Softmax iteration number was 500. Therefore, the training time was significantly reduced.

## 4. Methods 

### 4.1. Metabolomics Deep Belief Network

The DBN is a kind of deep neural network with multi-hidden layers that uses the restricted Boltzmann machines (RBM) method to complete the pretraining of the metabolomics data. The layers that are adjacent to each other are paired as an RBM, thus creating a two-layer cyclic neural network. There is no connection between the nodes of the same layer, and the nodes between the two layers have symmetric connections. The Boltzmann machine (BM) was first proposed in 1986 [34]. It has a high learning ability in the case of unsupervised learning. However, it requires high computational complexity. To solve this problem, RBM was proposed by Smolensky [35].

In the training of an RBM, *n* is the number of visible nodes and *m* is the number of hidden nodes. The input of RBM is the visible nodes, represented by vector *v*. The hidden nodes are the output, which is represented by vector *h*. If the current state is (*v*, *h*), the energy function formula is as follows [36]:

(1) $$E(v,h|\theta )=-\sum_{i=1}^{n}a_{i}v_{i}-\sum_{j=1}^{m}b_{j}h_{j}-\sum_{i=1}^{n}\sum_{j=1}^{m}v_{i}W_{ij}h_{j}$$

where *θ* includes the parameters *w_ij_*, *a_i_*, and *b_j_*—the metabolomic data feature states of visible unit *i* and hidden unit *j* are *v_i_* and *h_j_*, *a_i_* and *b_j_* are their biases, and *w_ij_* is the weight between them. For the metabolomic data, RBM training is implemented by optimizing *E*. The initial weight between the layers is obtained by RBM, and the outputs of each RBM are the inputs of the next RBM. When the metabolomic data features are extracted in an RBM, the activation function is used to calculate the outputs of the hidden unit and the reconstructed outputs of the visible unit. The activation function is the sigmoid function *δ* (*x*).

(2) $$\delta (x)=\frac{1}{1+e^{-x}}$$

The connection weights are obtained by the method of contrastive divergence (CD) learning with K (or CD-K) [36,37]. The updated formulae for the parameters are as follows:

(3) $$\Delta W_{ij}=\varepsilon (<v_{i}h_{j}{>}_{data}-<v_{i}h_{j}{>}_{recon})$$

(4) $$\Delta a_{i}=\varepsilon (<v_{i}{>}_{data}-<v_{i}{>}_{recon})$$

(5) $$\Delta b_{j}=\varepsilon (<h_{j}{>}_{data}-<h_{j}{>}_{recon})$$

where *ε* is the learning rate, Δ*W_ij_* and Δ*a_i_ (*Δ*b_j_*) are the updating values of weights and biases, respectively, respectively. <.>*_data_* and <.>*_recon_* represent the value of *v_i_* multiplied by *h_j_* before and after reconstruction, and the distribution of the reconstructed model can be reflected. The CD-1 learning strategy was chosen for reconstruction, and the reconstruction process was completed once.

### 4.2. Dropout

For large-scale data sets with high noise, deep learning has several advantages in feature extraction, classification, and recognition. However, deep learning is also prone to overfitting problems in small-scale training data sets [38]. In 2014, Hinton et al. [39] proposed a method called “dropout”. It randomly drops units (along with their connections) from the neural network during training. The dropout method samples for an exponential number of different “thinned” networks. These samples improve the performance of neural networks for the supervised learning tasks of many benchmark data sets. Wager et al. [40] analyzed dropout training as a form of adaptive regularization. This framework enabled them to uncover close connections among dropout training, adaptively balanced *L_2_*-regularization, and AdaGrad (an online optimization algorithm). This process led to a simple yet effective method for semi-supervised learning.

### 4.3. DBN + Softmax Regression

**Figure 4:**
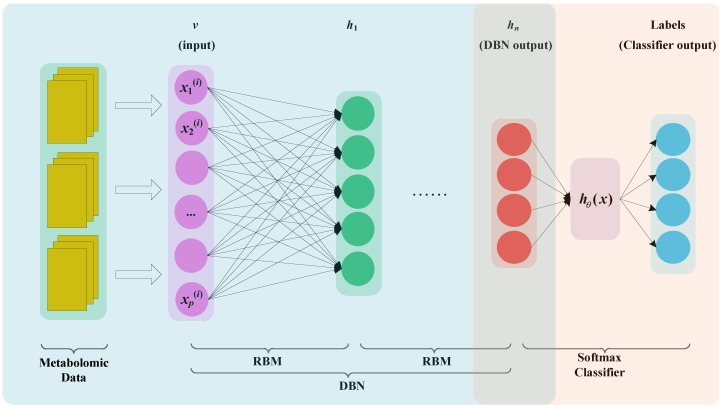
MDBN model. The yellow rectangles represent the metabolomics data. After preprocessing, the data are fed to DBN. The purple circles represent input layer. The green circles represent output layer of the first RBM. The red circles represent the output of DBN. The blue circles represent the output layer of Softmax classifier.

We established an end-to-end model that was a combination of the DBN and softmax regression to solve the metabolomic data classification problem. The MDBN model is shown in Figure 4. The input of the model was the metabolomic data obtained from biological experiments, and the output was a clinical diagnosis of BH. Metabolomic data consist of high-dimensional, sparse, and small samples. To avoid overfitting, a random number vector was generated during the process of pretraining and fine-tuning. A certain percentage of the vector is 0 or 1. After the output vector of the hidden layer unit is multiplied by the random number vector, the output value of the hidden layer unit is reserved or set to 0. The above process is the dropout process.

In Figure 4, the unlabelled data are used to pre-train the DBN model, and the labelled data are used to fine-tune the whole coherent system. The metabolomic data are the input layer *v*, and the output layer *h_n_* represents the features of metabolomics generated by DBN. The output layer *h_n_* is the input of softmax regression. Therefore, for the MDBN model, we first trained the DBN and then used the labelled data to train the softmax regression. This method can obtain the softmax regression initial weights instead of randomly generated weights, thereby completing the model training more effectively. Finally, after the whole system weights are initialized, fine-tuning optimization can be performed.

Softmax regression can be used for multi-classification problems, and is a further extension of logistic regression. In many areas, it has been used successfully to solve problems such as voice, video, and text classification. Metabolomic data are divided into training sets and test sets. Training data are used for model training and test data are used for model evaluation. In the softmax regression, if it is a *k* classification problem, the output of the softmax regression function is set to a *k* dimensional vector, and each dimension represents the probability that the inputs belong to each category. In the MDBN model, when the DBN training is completed, we used the labelled data to fine-tune all the parameters. During the fine-tuning stage, the model combined with DBN and softmax is considered to be the neural network, and the L-BFGS algorithm is used to optimize the global minimum for the cost function. The softmax regression and the system cost function are the same as in Ref. [41].

## 5. Conclusions

Metabolomic data are characterized by high-dimensional, sparse, and small samples. For the classification of BH, we collected the metabolomic data of female Wistar rats and introduced a framework MDBN, which combined a DBN and softmax regression. Dropout was added to prevent overfitting during the training processes. With the proposed method, we not only diagnosed BH but also completed an evaluation of RuXian-I. In the five-fold cross-validation experiment, the proposed model obtained better classification results than those of SVM, KNN, and BPNN, and the method was stable and reliable. To further verify the method proposed in this paper, the training set and test set data were divided into eight groups according to different proportions. We compared five models for these different groups of data. The experimental results show that our proposed framework is superior to those of other commonly used classification methods. 

## Figures and Tables

**Table 1:** Accuracies of classification in the positive spectrum data in the five-fold cross-validation experiment (%).

Group	BPNN	KNN	SVM	DBN+GD+Softmax	DBN+L-BFGS+Softmax
1	79.41	58.82	64.71	88.24	**94.12**
2	73.53	85.29	61.76	94.12	**97.06**
3	76.47	82.35	85.29	91.18	**97.06**
4	76.47	79.41	67.65	88.24	**91.18**
5	79.41	82.35	61.76	91.18	**97.06**
Mean	77.06	77.64	68.23	90.59	**95.30**

Bold values indicate the best results.

**Table 2:** Accuracies of the classification of negative spectrum data in the five-fold cross-validation experiment (%).

Group	BPNN	KNN	SVM	DBN+GD+Softmax	DBN+L-BFGS+Softmax
1	94.12	97.06	**100.00**	**100.00**	**100.00**
2	88.24	79.41	94.12	**100.00**	94.12
3	91.18	91.18	82.35	**94.12**	**94.12**
4	91.18	94.12	61.76	**97.06**	82.35
5	73.53	79.41	52.94	73.53	76.47
Mean	87.65	88.24	78.23	**92.94**	89.41

Bold values indicate the best results.

**Table 3:** Classification accuracies of different training and test sets (%).

Training Set	Test Set	BPNN	KNN	SVM	DBN+GD+Softmax	DBN+L-BFGS+Softmax
50	118	59.32	33.05	39.83	**72.03**	66.95
60	108	68.52	70.37	40.74	77.78	**83.33**
70	98	74.49	83.67	40.82	**83.67**	**83.67**
80	88	84.09	84.09	46.59	92.05	**94.31**
90	78	78.21	82.05	48.72	88.46	**92.31**
100	68	77.94	79.41	44.12	89.71	**92.65**
110	58	77.59	75.86	53.45	91.38	**93.10**
120	48	81.25	70.83	54.17	89.58	**91.67**

Bold values indicate the best results.
